# Feasibility of assessing utilities with a single-item standard gamble questionnaire in patients with melanoma

**DOI:** 10.1186/s41687-021-00350-w

**Published:** 2021-08-28

**Authors:** Christine Blome, Katharina C. Kähler, Tobias Wagner, Axel Hauschild, Matthias Augustin

**Affiliations:** 1 Institute for Health Services Research in Dermatology and Nursing (IVDP), University Medical Center Hamburg-Eppendorf (UKE), Martinistraße 52, 20246 Hamburg, Germany; 2 Department of Dermatology, Venerology and Allergology, University Hospital Schleswig-Holstein (UKSH), Kiel, Germany

## Abstract

**Objectives:**

To determine the feasibility of eliciting utilities with a standard gamble self-completion questionnaire that uses a single-item approach in melanoma patients.

**Methods:**

150 patients with low-risk melanoma completed a paper standard gamble questionnaire. Six scenarios described the adjuvant treatment of high-risk melanoma with interferon alfa-2b with varied side effects. Patients were asked to directly state the maximum death risk they would accept to prevent these health states. Methods were the same as in a study by Kilbridge et al. (J Clin Oncol 19(3):812–823, 2021. 10.1200/JCO.2001.19.3.812), except that they used computerised interviews and an iterative risk variation (Ping–Pong method) to elicit utilities.

**Results:**

The rate of missing values in the standard gamble was 1.0%. The percentage of patients who misordered scenarios was very similar to the reference study (11.3% vs. 11.2%). Mean utilities were also similar with a maximum difference of 0.02 points, but median utilities were not (between 0.21 points below and 0.05 points above the reference study).

**Conclusions:**

One-item utility elicitation with questionnaires might be a feasible alternative to computerised face-to-face interviews to conduct a standard gamble in melanoma patients.

## Introduction

Standard gamble (SG) is a method used to determine the utility of health states, which is needed for health-economic evaluations. SG asks participants to choose between two hypothetical options: either living with a certain health state of interest or taking a risky treatment with a success chance *p* of full health (or being disease-free) and a complementary chance (1−*p*) of instant painless death [1]. The health state to be rated can be either the respondent’s own current health or a health state scenario. SG mostly uses iterative variation of the death risk, either by gradually decreasing the chance *p* of full health while gradually increasing the chance 1−*p* of death (top-down titration) or by moving back and forth between higher and lower values (e.g., 100%, 10%, 90% etc., Ping–Pong method [2]).

SG can be administered with in-person interviews or in a self-completion format (computerised or on paper). Administering the SG with self-completion paper questionnaires [3, 4] can be less time-consuming and cost-intensive than in-person interviews. The paper SG [3, 5], for example, uses top-down titration; when used to rate the participants' own current health, it showed high concordance with a computer-based self-completion SG [3]. However, in another study, a mailed paper-based SG with top-down titration was not regarded feasible because many patients obviously had difficulties understanding the task [4].

Instead of presenting a series of choices, utility has also been assessed with a single question per health state. Here, participants directly stated the minimum chance of success (vs. risk of death) a treatment should have so that they would accept it. Concordance between this approach and a computerised SG using the Ping–Pong method was low in a sample of depressive patients, but it remained unclear if this actually resulted from the single-item assessment [6].

Given these inconclusive findings, we aimed to assess the feasibility of conducting a SG with a self-completion paper questionnaire using a single utility item in melanoma patients.

## Methods

We replicated a study on preferences of melanoma patients towards adjuvant interferon alfa-2b (IFN-α 2b) treatment with varied toxicity profiles [7]. While the original study used an in-person computerised SG interview and the Ping–Pong method, we used an SG paper questionnaire with one-item utility assessment. The main aim of the study was to determine the utility of different treatment scenarios (these results have been reported in detail elsewhere [8]); here, we report on the feasibility of the utility assessment.

Participants had an excised low-risk melanoma diagnosed at least eight weeks before and had received definitive surgical therapy for melanoma with no adjuvant treatment planned. As in the reference study [7], the reason for including low-risk instead of high-risk melanoma patients was that participants should be familiar with melanoma but should not be influenced in their decisions regarding adjuvant treatment. Patients were recruited in ten German dermatological centres and gave informed consent. They completed the questionnaire in the dermatological centres while waiting for their appointment, but without clinical staff being present.

The questionnaire structure was as follows. After a short introduction to study aims and basic information on malignant melanoma, two test SG scenarios on monocular and binocular blindness were rated. Then, IFN-α 2b treatment in general was described, followed by SG for six scenarios. The remainder of the questionnaire collected data on attitudes towards the study, socio-demographic data, and others (not reported here).

**Fig. 1:**
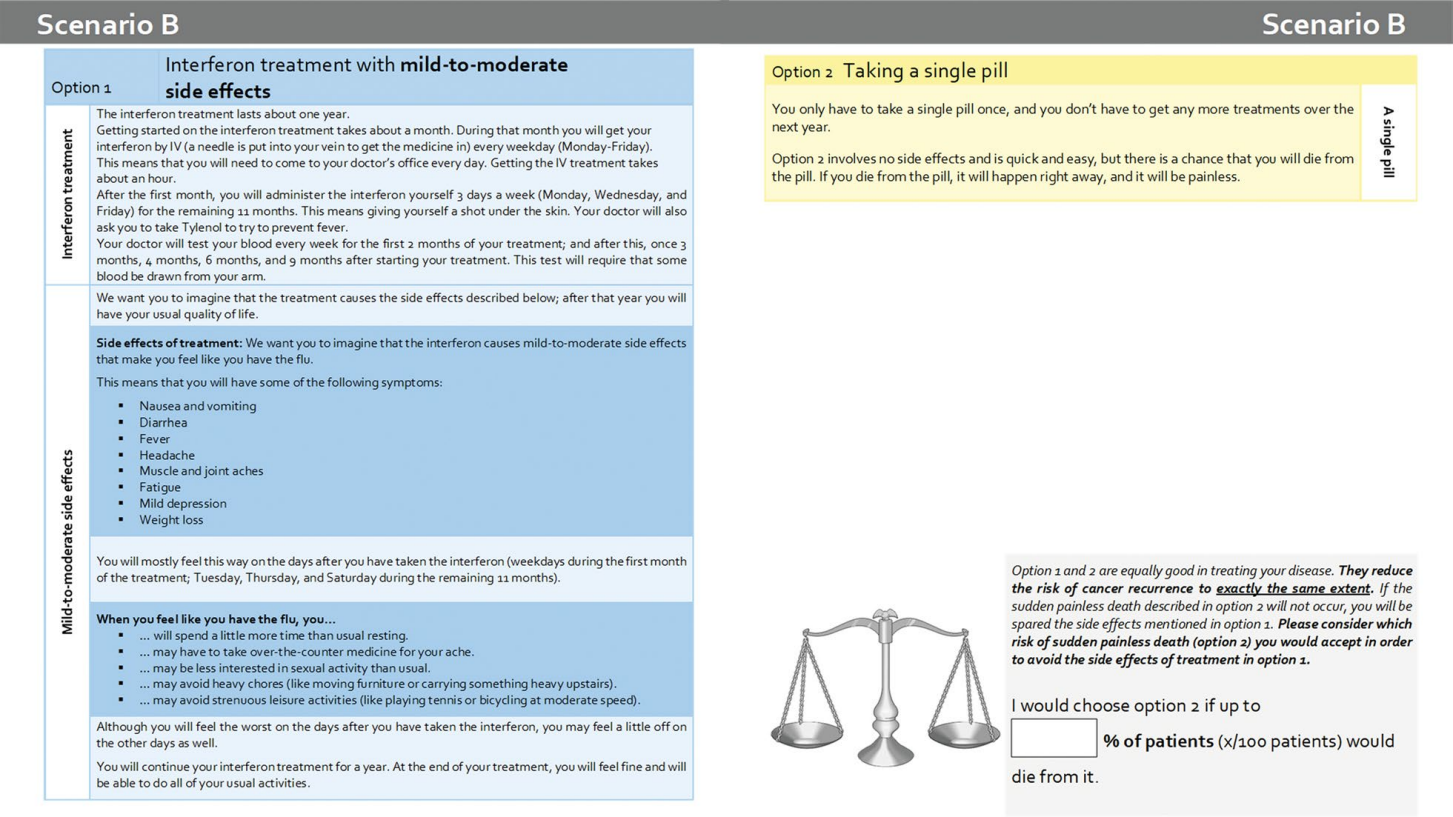
Example scenario with single-item standard gamble question (own translation from German original questionnaire)

The six scenarios were: IFN-α 2b treatment for one year (A) without side effects (but only the inconvenience of visits to the physician, medication, and blood tests); (B) with mild-to-moderate side effects (Fig. 1); (C) with mild-to-moderate side effects and laboratory side effects (leading to dose reduction and, in three out of ten patients, to termination of treatment); (D) with severe side effects (e.g., flu-like symptoms, fatigue, weight loss); (E) recurrence and death from melanoma after previous IFN-α 2b treatment (with mild impairments at first, followed by severe impairments leading to death); (F) recurrence and death from melanoma without previous IFN-α 2b treatment (with mild impairments at first, followed by severe impairments leading to death).

Each SG was presented on a double page with the scenario at the left and the alternative on the right side. In order to facilitate understanding, differences to the preceding scenario were highlighted in scenarios B–D, and scenario subsections were labelled with keywords in the margins (e.g., severe side effects). As an example, scenario B is presented in Fig. 1. The choice question was (here for scenario B): "Option 1 and 2 are equally good in treating your disease. They reduce the risk of cancer recurrence exactly to the same extent. If the sudden painless death described in option 2 will not occur, you will be spared the side effects mentioned in option 1. Please consider which risk of sudden painless death (option 2) you would accept to avoid the side effects of treatment in option 1. I would choose option 2 if up to ____% of patients (x/100 patients) would die from it."

## Results

Overall, 174 patients agreed to participate, 150 of whom completed and returned the questionnaire (86.2%). Of these, 20 patients (13.3%) were excluded from analysis: three (2.0%) did not provide socio-demographic data or did not respond to any SG scenario, and 17 patients (11.3%) misordered scenarios (for example by assigning higher utility to being blind on two eyes than to being blind on one eye), indicating a lack of understanding of the SG task. In the reference study [7], 11.2% of participants were excluded for disorder of scenarios. Since no missing values were reported, we assume there were none due to the face-to-face data assessment.

Almost half of the patients (47.3%) were female, mean age was 54.4 years (range 25–82, median 53), and 82.3% had an intermediate secondary education or higher.

Between 0 and 3 patients did not respond to single scenarios (Table 1); the rate of missing values across all six scenarios was 1.0% (8 in 780 possible responses missing). In the reference study [7], there were no missing values in the six scenarios, most likely due to the forced-choice computerised interview [9].

**Table 1:** Standard gamble utilities (range 0 = dead 1 = health state without treatment side effects or recurrence of melanoma) for health states in our study and in Kilbridge et al. [7]

Scenario	Scenario summary	This study, n = 130				Kilbridge et al. (2001), n = 95			
		Mean	SD	Median	% Missing	Mean	SD	Median	% Missing
(A) IFN-α 2b treatment without side effects	Treatment involves: 1 month of daily intravenous IFN administration; 11 months of 3 self-administrations of IFN per week; medication for fever prevention; 12 blood tests. No side effects	0.94	0.14	0.99	1.5	0.92	0.15	0.98	0.0
(B) IFN-α 2b treatment with mild-to-moderate side effects	Treatment as in scenario A. Side effects similar to a flu (e.g., nausea, headache, fatigue) leading to impairments such as spending a little more time resting	0.90	0.18	0.99	1.5	0.88	0.17	0.97	0.0
(C) IFN-α 2b treatment with mild-to-moderate side effects and laboratory side effects	Treatment as in scenario A. Side effects as in scenario B. Serious abnormalities in blood tests leading to dose reduction and possibly to termination of treatment without reduction of effectiveness	0.88	0.20	0.99	0.8	0.86	0.20	0.94	0.0
(D) IFN-α 2b treatment with severe side effects	Treatment as in scenario A. Side effects as in scenario B, but with at least one episode of severe side effects similar to a bad flu (e.g., very high fever, frequent vomiting, depression and suicidal ideation) leading to impairments such as inability to do even light chores. Dose reduction and possibly termination of treatment without reduction of effectiveness	0.81	0.25	0.90	0.0	0.81	0.25	0.90	0.0
(E) Recurrence and death from melanoma after previous IFN-α 2b treatment	Recurrence of melanoma after having completed 12 month treatment. Symptoms lead to impairment such as spending a little more time resting. Later, symptoms increase progressively, leading to death from melanoma	0.60	0.32	0.50	1.5	0.62	0.34	0.71	0.0
(F) Recurrence and death from melanoma without previous IFN-α 2b treatment	As in scenario E, but without having received previous treatment for melanoma	0.60	0.31	0.50	2.3	0.61	0.34	0.67	0.0

*IFN-α 2b* interferon alfa-2b, *SD* standard deviation

With regard to attitudes towards the study, 22.3% of our patients agreed with the statement "I was upset by answering the questions in this questionnaire", as compared to 15.0% who agreed with "I was upset by this interview" in the reference study. A total of 67.7% agreed with "My answers do a good job showing how I feel about different health conditions" (reference study: 97.2%), 86.4% agreed with "These questions made me think hard about my personal values, preferences, and feelings" (reference study: 96.3%), and 80.0% agreed that "This study could help doctors better understand how patients feel about their health" (reference study: 97.2%).

**Fig. 2:**
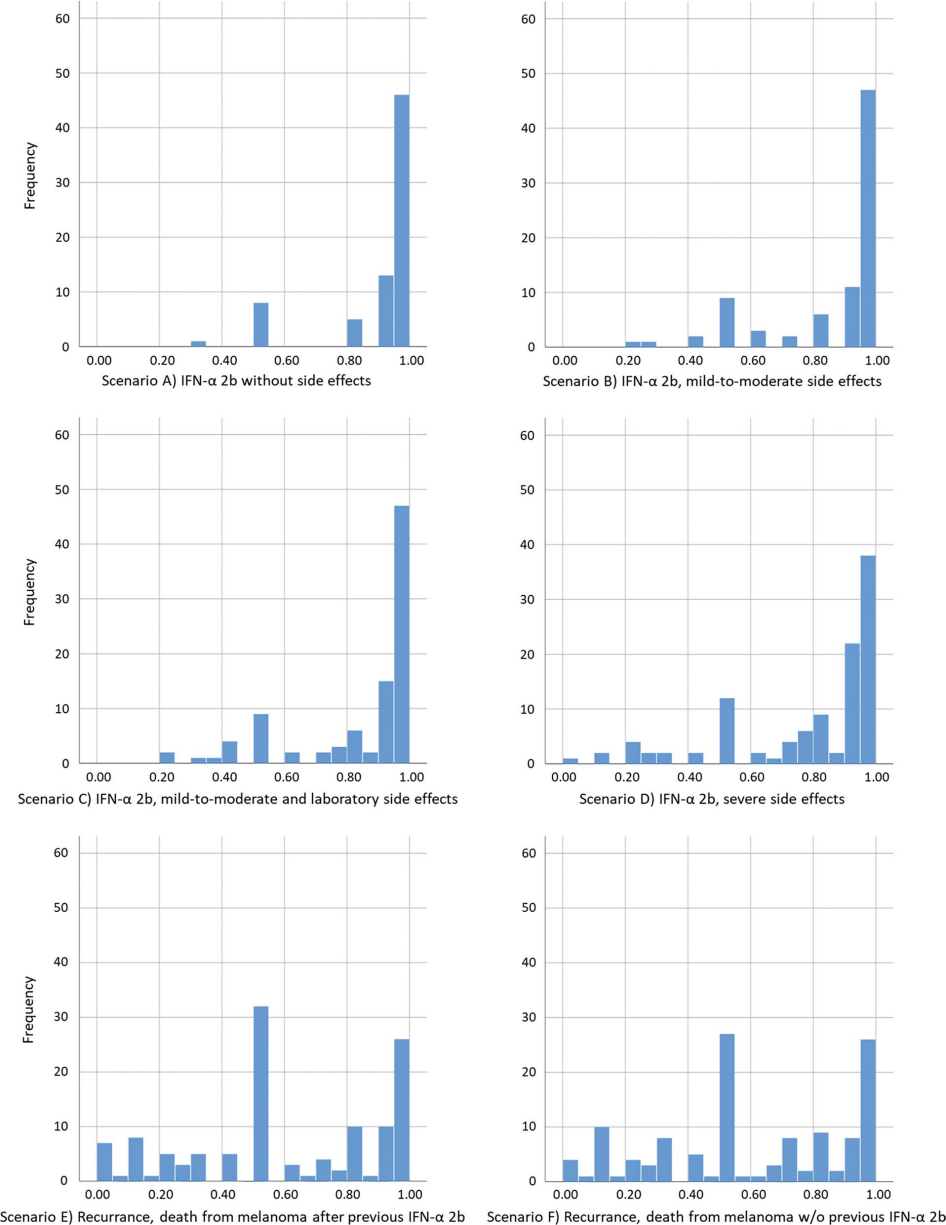
Distribution of standard gamble utility ratings by scenario

As expected, utility was highest for scenario A (no side effects) with an average of 0.94 and decreased with the intensity of side effects described in scenarios B–D (Table 1). Recurrence and death from melanoma had the same utility of 0.60 either with (scenario E) or without (scenario F) previous IFN-α 2b treatment. These mean utilities were very similar to those found by Kilbridge et al. [7] with a maximum of 0.02 points difference. However, differences between median values in the two studies were higher, especially in scenarios E and F where we found a median utility of 0.50 each as opposed to 0.71 and 0.67 in the reference study. Figure 2 shows the distribution of utility ratings, which were considerably more heterogeneous in scenarios E and F than in A–D.

## Discussion

We assessed utilities for IFN-α 2b toxicities with questionnaires instead of computerised interviews, and we used a one-item utility assessment instead of iterative probability variation. Nevertheless, patients seemed to manage the difficult task of the SG well: The rate of patients who misordered scenarios was very similar to the reference study with 11.3% vs. 11.2% (which of course does not imply that the remaining patients in both studies necessarily understand the SG task correctly). The mean utilities were also quite similar. This convergence was all the more surprising as there are further differences between the two studies, with the reference study being conducted in the U.S. instead of Germany and more than a decade earlier. However, median utility values were less similar, and a higher proportion of patients felt upset by the study and a lower proportion of patients considered their responses informative and well thought through. The latter may either indicate that patients indeed feel more comfortable with a SG when conducted in a face-to-face interview. Alternatively, the difference could be due to social desirability bias, with people being more hesitant to criticise the study in a face-to-face interview than in a questionnaire. The high proportion of participants who stated feeling upset by the questions in both studies may point at a general problem with the SG approach which asks respondents to hypothetically trade the risk of instant death against impaired health. Another reason may be that participants were confronted with information on side effects of melanoma treatment and the possibility of melanoma recurrence; this may have been perceived as threatening, even though our participants had experienced low-risk melanoma only.

In this study, 47% of patients were female, which reflects the gender distribution in patients with melanoma in Germany [10]. However, while the median age at melanoma diagnosis is 64 years, our sample was younger with a median age of 53 [10].

As a limitation, we could only compare our results with the in-person computerised SG interview used in the reference study, but not with other assessment methods such as computerised self-completion SG without interviewers or non-computerised in-person interviews. We also do not know whether participants understood the SG task correctly in both our and the reference study, even if they had no missing values and did not misorder scenarios.

It should also be noted that in this study, a utility of 1 does not equal full health but the health state without treatment side effects or recurrence of melanoma as described in the scenarios. This is because the second option (the pill) is described as preventing the respective scenario, but not as preventing any other health impairment. Utilities found in this study are therefore not comparable with utilities ranging from “dead” to “perfect health” without adjustment [11]. In addition, we did not allow for scenarios to be rated worse than death, which would lead to negative utility values. As both are also true for the reference study, this does not impair the comparison between the two approaches that this manuscript targets but should be considered in future uses of the paper-based one-item approach.

In the reference study, patients were presented with both chance of survival (*p*) and death risk (1−*p*) of the treatment. In our study, we had to decide whether to ask for *p* or 1−*p* because patients should provide a specific number instead of deciding for one out of two options. We chose to ask for 1−*p* (risk of death) for two reasons. One, this means that both options are framed in the same negative direction (inconvenience, side effects, symptoms vs. risk of death). Two, we felt that this allowed for a more comprehensible SG question. However, had we asked for the minimum chance of survival instead, patients may have been willing to accept a riskier treatment, as positive framing is associated with the treatment being perceived as less harmful [12], resulting in lower utility values.

In conclusion, the paper questionnaire-based one-item utility elicitation used in this study resulted in very similar mean (but not median) utility values as the computerised face-to-face interviews in the reference study [7]. It may be a feasible—and cost-saving—alternative in situations where interview and/or computerised SG is difficult, for example if patients shall be reached by mail over a large geographic area and for patients who are not computer-literate or do not have access to a computer. Thus, further research on the reliability and validity of this approach is warranted.

## Data Availability

The datasets used and/or analysed during the current study are available from the corresponding author on reasonable request.
